# Management of Hypothyroidism in Patients with Acute Myocardial Infarction

**DOI:** 10.3390/medicina56050214

**Published:** 2020-04-28

**Authors:** Danielle Eagan, Gabriela Spencer-Bonilla, Spyridoula Maraka, Monica Aggarwal, Naykky Singh Ospina

**Affiliations:** 1 Division of Endocrinology, Diabetes, & Metabolism, University of Florida, Gainesville, FL 32610, USA; 2 Department of Medicine, Stanford University School of Medicine, Stanford, CA 94305, USA; 3 Division of Endocrinology and Metabolism, Center for Osteoporosis and Metabolic Bone Diseases, University of Arkansas for Medical Sciences and the Central Arkansas Veterans Health care System, Little Rock, AR 72205, USA; 4 Division of Cardiovascular Medicine, University of Florida, Gainesville, FL 32610, USA

**Keywords:** hypothyroidism, levothyroxine, myocardial infarction, subclinical hypothyroidism, cardioprotection

## Abstract

*Background and Objectives*: Thyroid hormones (TH) affect cardiac function through effects on cardiac contractility and systemic vascular resistance. While TH replacement for patients with hypothyroidism might be necessary for restoration of cardiac output after an acute myocardial infarction (AMI), it could theoretically lead to excessively rapid restoration of the metabolic rate. The appropriate management of hypothyroidism in patients with AMI is unknown. We describe the practice patterns in the management of hypothyroidism in the setting of AMI as well as patients’ clinical outcomes. *Material and Methods*: Retrospective study of patients that were admitted to a tertiary care hospital with AMI and newly diagnosed or uncontrolled hypothyroidism (TSH ≥ 10 mIU/L) between 2011–2018. Eligible patients were identified using diagnosis codes for AMI and laboratory values, followed by medical record review. We categorized patients according to treatment status with TH and by degree of hypothyroidism. Clinical outcomes included: 30-day mortality/readmission, bleeding, stroke, arrhythmia, sudden cardiac death, and new or worsening heart failure. Summary statistics and group comparisons are presented. *Results*: Sixty-four patients were included, their median age was 64 years and 61% (*n* = 39) were women. Most of the patients (59%) had a documented history of hypothyroidism. Of these, all were restarted on levothyroxine (LT4) during the index admission when compared to patients without a history of hypothyroidism, of which 54% received LT4 treatment (*p* = 0.001). The median TSH in those treated with LT4 was higher (25 mIU/L) when compared to those who were not (12 mIU/L), (*p* = 0.007). Patients who received intravenous LT4 had higher TSH levels and other variables suggesting worse clinical presentation, but these differences were not statistically significant. No statistically significant differences were noted on clinical outcomes according to LT4 treatment status. *Conclusion*: A history of hypothyroidism and the degree of TSH elevation seem to guide the management of hypothyroidism in patients with AMI. The clinical effect of correcting hypothyroidism in this setting requires further evaluation.

## 1. Introduction

Thyroid hormones (TH) affect the function of the cardiovascular system by modulating genomic and non-genomic pathways in cardiac myocytes, vascular smooth muscle, and endothelial cells, and by their effects on lipid metabolism and other inflammatory pathways that are associated with cardiovascular disease [1,2]. In patients with hypothyroidism, the proposed negative effects on the cardiovascular system include increased systemic vascular resistance and decreased cardiac output, heart rate, and myocardial contractility [1,2,3].

The physiological relationship between the cardiovascular system and thyroid function has led to multiple observational studies evaluating the association between hypothyroidism and cardiovascular disease, which have described higher risk of cardiovascular events and mortality in patients with hypothyroidism as compared to those who were euthyroid [4,5]. Low triiodothyronine (T3) has been identified as a prognostic factor for patients with acute myocardial infarction (AMI) associated with increased biomarkers of cardiac injury, decreased left ventricular function, and increased mortality [6,7].

A smaller number of studies have investigated the effect of levothyroxine (LT4) treatment and correction of hypothyroidism on clinical important outcomes, suggesting a benefit for those with overt hypothyroidism (OH) and inconsistent results for those with subclinical hypothyroidism (SCH) [8,9,10,11,12]. OH is characterized by low circulating levels of thyroxine (T4) associated with a compensatory elevation of thyroid stimulating hormone (TSH) levels [13,14,15]. SCH, which affects a larger proportion of the population (3–9%), is a biochemical diagnosis that is defined by normal levels of T4 associated with TSH levels above the upper limit of normal [1,13,14,15].

Most studies have focused on the association of hypothyroidism and cardiovascular disease in the chronic and outpatient setting. However, the physiological relationship between these two systems could have clinical implications in the acute setting and affect a large number of individuals. For example, a prospective study including 400 patients without previous LT4 treatment admitted for management of acute coronary heart disease found that, although most of the patients were euthyroid (77%), 8% had OH and 3% SCH [16]. A larger multicenter study, including 1806 patients, found a prevalence of SCH of 17%, after excluding 6% of patients who were on TH replacement [17]. 

The ideal management of patients found to have biochemical hypothyroidism in the setting of AMI is complex, due to physiological changes that occur in response to acute illness and limited clinical evidence regarding the risk and benefits of treatment. In the acute state, most patients are found to have a downregulation of TH that seems to occur rapidly, usually in the first few days after the event [18]. In fact, 5–35% of patients with an AMI are found to have low T3 syndrome (low T3 concentrations that are associated with normal/mildly reduced serum T4 and TSH levels and increased reverse T3 levels) [6,17,19,20].

Clinical evidence guiding the management of hypothyroidism in the setting of AMI is scarce and no specific recommendations are available. We describe the clinical presentation, thyroid replacement practice patterns, and outcomes of patients found to have hypothyroidism in the setting of an AMI to address this knowledge gap.

## 2. Materials and Methods

### 2.1. Study Design

We retrospectively analyzed the electronic medical records of patients that were admitted with AMI and found to have TSH levels ≥ 10 mIU/L at the University of Florida (UF) between 2011 and 2018. This study was approved by the Institutional Review Board (IRB) of UF under IRB201900380 approved on 11 February 2019 as exempt, because it posed a minimal risk to participants. Under this approval, a waiver of consent was provided to conduct research on previously collected clinical information.

### 2.2. Study Population

**Figure 1:**
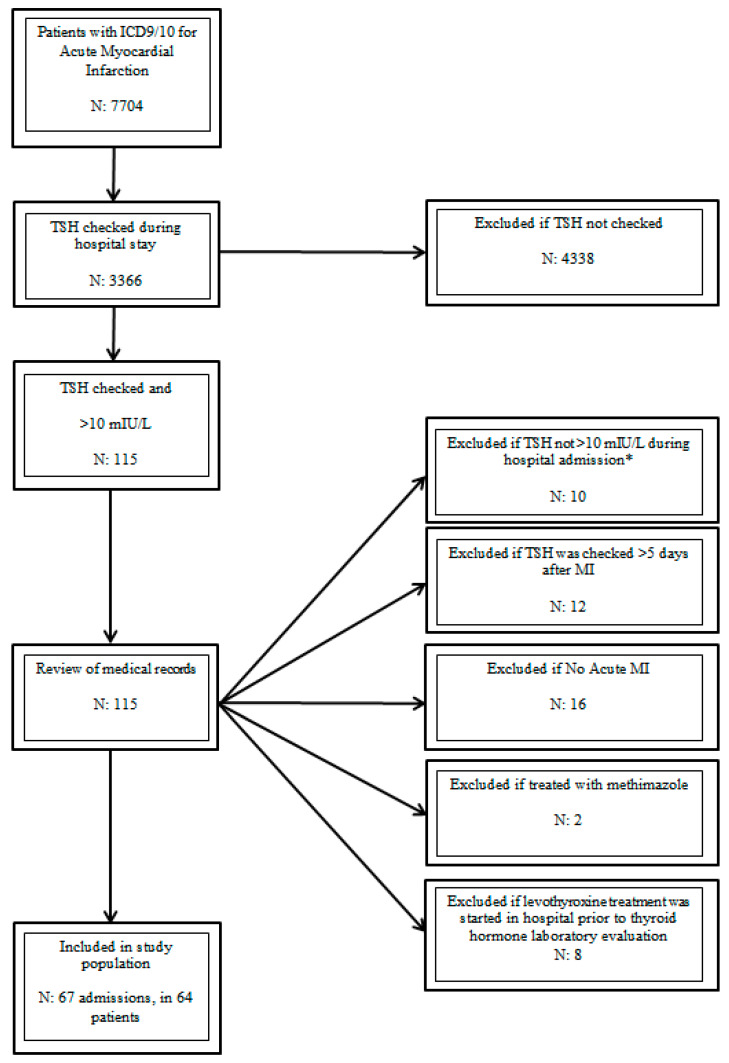
Study Population. ICD (International Classification of Disease), TSH (thyroid stimulating hormone), and MI (myocardial infarction). * Led to exclusion of patients given that they did not have a baseline TSH ≥ 10 during a AMI admission.

Patients with AMI were initially identified within the UF Health integrated data repository while using International Classification of Disease (ICD) 9/10 codes (Appendix A) and laboratory values. The medical records of this initial cohort were further evaluated to: 1) verify the diagnosis of AMI based on clinical presentation, electrocardiogram changes, and laboratory abnormalities and 2) the presence of an elevated TSH (≥10 mIU/L) measured within five days of the AMI and before any inpatient treatment with TH was initiated. As a result, patients with newly diagnosed hypothyroidism or those with uncontrolled/undertreated hypothyroidism at the time of evaluation were included. Patients who received inpatient treatment with thyroid hormones before measurement of thyroid function were excluded. The patients who received active treatment of hyperthyroidism were also excluded. For patients admitted more than once, the first admission was used as the index admission (Figure 1).

### 2.3. Study Measurements/Assessments

An electronic data collection form was developed and standardized following a data dictionary to define the variables of interest. Demographics, comorbidities, risk factors for cardiovascular disease, physical exam, laboratory and imaging findings, thyroid dysfunction history, AMI presentation, management, and outcomes were recorded. The risk for myxedema coma was also evaluated using a validated risk score [21]. The patients were classified according to: (1) thyroid function status on admission and (2) treatment with TH during the hospital admission.

Based on the initial TH evaluation, patients were categorized as having: (1) “OH” if TSH was ≥10 mIU/L and free T4 levels were below the lower limit of the reference range, (2) “SCH” if TSH was ≥10 mIU/L and free T4 levels were within the reference range, and (3) “high TSH” if no free T4 measurement was available, but the TSH was ≥10 mIU/L. Patients with SCH and high TSH were analyzed together. The patients were classified as treated if TH replacement was started during the hospital stay and not treated if no replacement was initiated. Clinical outcomes of interest included 30-day mortality, 30-day readmission, and new or worsening heart failure. We also evaluated the development of bleeding, stroke, or arrhythmia during the hospital stay according to treatment status and timing of TH administration (before/after) for those who were treated.

### 2.4. Thyroid Hormone Assays

During the study period, the TSH measurements were carried out by immunoassay using the Beckman Coulter Dxl, with reference range of 0.27–4.2 mIU/L and 0.4–5 mIU/L. The free T4 levels were measured similarly, with reference range of 0.93–1.7 ng/dL, 0.8–1.7 ng/dL, and 0.6–1.2 ng/dL.

### 2.5. Data Analysis

Statistical analyses were performed using JMP [22]. Descriptive summary statistics according to variable type are presented; frequencies were used for categorical variables and mean ± standard deviation or median/interquartile range (IQR) for continuous variables according to distribution. The differences between categorical variables were assessed using the χ^2^ test or the Fisher’s exact test and differences between continuous variables using the independent *t*-test or Mann–Whitney test as appropriate. A *p* value of <0.05 was considered to be statistically significant.

## 3. Results

An initial cohort of 115 patients admitted with a diagnosis of AMI and found to have a TSH ≥10 mIU/L was identified. After review of medical records, 64 patients with 67 admissions fulfilled the inclusion criteria (Figure 1).

### 3.1. Demographics, Thyroid Status and Treatment of Hypothyroidism

The median age at the time of admission was 64 years. The majority of patients were women (61%) and 78% of the population was Caucasian. The most common risk factors for cardiovascular disease included hypertension (70%), dyslipidemia (66%), and diabetes (45%). A history of coronary heart disease was present in 42% of the patients and 31% had history of heart failure. 

Biochemical evaluation showed SCH in 47% (*n*, 30) of the patients, OH in 37% (*n*, 24), and elevated TSH level without a free T4 measurement in 16% (*n*, 10). Most patients (84%) presented with non-ST elevation acute myocardial infarction (NSTEMI) and 16% with ST elevation myocardial infarction (STEMI).

In terms of treatment, 52 patients received LT4 treatment (81%). Most of the patients who received LT4 therapy were started within 72 h of their AMI presentation (83%). The median time to LT4 treatment was two days (IQR 1–3).

All of the patients treated with LT4 were treated with generic or brand name levothyroxine (IV or oral), no gel or liquid formulations were used. No patient was treated with T3 therapy. Table 1 provides demographics and risk factors according to thyroid and treatment status.

### 3.2. Clinical Presentation, Laboratory and Imaging Results

In most patients (67%), the TSH was checked within 24 h of the AMI, with a median time to TSH measurement of one day (IQR 1–2). A free or a total T3 was checked in 18 patients (28%) and 72% had values below the lower limit of normal.

The median initial TSH level was 20 mIU/L, with higher values in the OH group (42 vs. 14 mIU/L). Patients who received treatment had a higher TSH (25 mIU/L, IQR, 13–43) when compared to those who did not receive LT4 treatment (12 mIU/L, IQR, 11–19), (*p* = 0.007). In the group of patients that were not treated with LT4, 50% were hemodynamically unstable at baseline when compared to the 21% of those who were treated. No other statistically significant differences were found when comparing the clinical presentation of patients according to the thyroid function and treatment status (Table 2).

### 3.3. Thyroid History and Hypothyroidism Management

Documentation of previous treatment for hypothyroidism was found in 38 out of 64 patients before admission (59%), only one of these patients was on treatment with both T4/T3 and the rest received LT4 treatment only. The median pre-admission LT4 dose was 100 mcg daily (63–125 mcg) with a median dose of 1.3 mcg/kg (0.8–1.8 mcg/kg). All patients with a previous documentation of hypothyroidism treatment were treated with LT4 during the hospital admission, while 54% of those without a history of hypothyroidism were started on replacement, (*p* < 0.001). Out of the 38 patients with previous documentation of hypothyroidism, 34 had documentation of the previous LT4 dose. The home dose was continued on 70% of the patients, decreased in 18%, and increased in 12%.

Most patients had a previous history of hypothyroidism (59%) and their median TSH was 23 (13–38) mIU/L. All patients in this group received treatment with LT4 (15/38 had overt hypothyroidism). A new diagnosis of hypothyroidism was found on 41% of the patients. Their median TSH was 14 (12–45) mIU/L. Nine patients had overt hypothyroidism in this group and 8/9 were treated (89%).

Endocrinology was consulted in 27% of the admissions. More patients with OH received treatment (96%) when compared to those with SCH/high TSH (73%), (*p* = 0.0230). In patients in which treatment was started/resumed, 85% were treated with oral LT4, with similar rates in the OH group (83%) and the SCH/high TSH group (86%). The median initial oral dose was 100 mcg (50–125) of LT4 with a median of 1.3 mcg/kg (0.7–1.7). Eight patients (15%) received intravenous (IV) LT4 with an initial median IV dose of 63 mcg (31–100) and 0.9 mcg/kg (0.6–1.1).

The percentage of patients who had a myxedema score ≥60, EF ≤ 40%, and were hemodynamically unstable was higher in those that received IV LT4 when compared to those that received oral LT4. The median TSH was also higher in those who received IV LT4. These differences were not statistically significant (Table 3).

### 3.4. MI Management

During the hospital admission, all patients received treatment with anticoagulants and most patients received treatment with antiplatelet, beta-blockers, angiotensin-converting-enzyme inhibitors (ACEI), and statins. More patients who received treatment with LT4 had treatment with ACEI. Percutaneous coronary intervention was performed in 28% of patients and 5% underwent coronary artery bypass grafting. No other differences in the management of the AMI were noted (Table 4).

### 3.5. Clinical Outcomes

Six patients (9%) died within 30 days of admission from direct complications of the AMI or infection. Five of these patients died during their index admission and one patient was discharged and died at home. In addition, one patient died on day 53 of his hospital admission, leaving 58 patients who were discharged from the hospital and were included in the readmission analysis. The rate of 30-day mortality was lower in those who were treated (6%) when compared to those not treated (25%) with LT4. Similarly, the rate of new or worsening heart failure was higher (42%) in those who did not receive LT4 treatment for hypothyroidism when compared to those treated (25%). Thirteen percent of the patients who received LT4 treatment were readmitted (five due to direct complications of the AMI and one due to infection) and none of the untreated patients were readmitted. These differences were not statistically significant. No patient was found to have sudden cardiac death. Table 5 presents a distribution of outcomes according to treatment and thyroid function status.

The proportion of patients who developed bleeding, stroke, or arrhythmia during the hospital stay was similar between those who were treated and those untreated with LT4. The percentage of patients who developed an arrhythmia requiring clinical intervention was 33% in the group of patients who did not receive treatment as compared to 23% for those who received LT4 treatment; however, no statistically significant differences were noted. Table 6 notes a distribution of outcomes according to treatment status and time of the event as it relates to LT4 therapy.

## 4. Discussion

In this retrospective study of patients admitted for the management of AMI and found to have biochemical evidence of hypothyroidism (TSH ≥ 10 mIU/L), patients with higher TSH values and previous history of hypothyroidism were more likely to receive LT4 in the acute setting. In most patients, treatment with oral LT4 was prescribed and, in those with previous history of hypothyroidism, the dose was rarely increased, despite elevated TSH levels. Although we did not find a statistically significant difference, more patients treated with IV LT4 had a myxedema score ≥ 60, EF ≤ 40% or were hemodynamically unstable (suggesting worse clinical presentation) when compared to those that were treated with oral LT4. 

In terms of clinical outcomes, patients who were not treated with LT4 had a higher rate of 30-day mortality and new/worsening heart failure, but a lower rate of readmission. However, these differences did not reach statistical significance, which was possibly due to our small sample size versus a true lack of difference in effect. Similarly, the rate of bleeding, stroke, and arrhythmia was not statistically different between the treatment groups.

We found that a previous history of hypothyroidism and the degree of TSH elevation are important factors guiding the management of hypothyroidism in the setting of AMI. Clinical guidelines recommend the evaluation of thyroid function status for patients at high risk of hypothyroidism or with known hypothyroidism during any hospital admission to guide the need to restart and/or modify their TH replacement regimen. Moreover, the oral route is preferred for replacement, with the goal of long-term normalization of TH levels. For patients in whom the oral route is not feasible, those with malabsorption, or with myxedema coma, the IV route should be considered [23]. Similarly, clinical practice recommendations suggest a low starting dose and the careful titration of LT4 treatment for elderly patients and those with cardiac disease, given concerns that the inotropic and chronotropic effects of LT4 therapy could increase the metabolic demands and unmask previously compensated ischemic disease, even in the non-acute setting [3,23]. Although these clinical practice recommendations are not specific for patients with AMI, our findings suggest that, in the absence of direct clinical evidence, clinicians follow these recommendations (low dose, oral replacement), without any statistically significant difference in terms of clinical outcomes between treatment groups. In fact, despite evidence of hypothyroidism, the home dose was unchanged or decreased for most patients with previous history of hypothyroidism, suggesting caution by clinicians when restarting therapy in the setting of an AMI. A study that evaluated 133 patients who were admitted to the intensive care unit for more than seven days found that ~17% had their LT4 therapy withheld, which suggested that the prescription of chronic therapy (in this case TH) might be inadequate during hospital admissions [24]. However, in our study, TH replacement was restarted for all patients with previous documentation of hypothyroidism. 

There is an ongoing debate regarding whether TH changes during critical illness are beneficial or maladaptive and whether they can mostly provide prognostic information or are a signal that immediate treatment is needed. Further evaluation is required in the specific case of hypothyroidism and AMI, as clinical evidence guiding adequate diagnosis and management is scarce.

In terms of diagnosis, the response changes in the thyroid axis as a reaction to acute illness can limit the ability of thyroid testing to assess thyroid function and guide management decisions. The timing of the TSH sample as it relates to the AMI and time of the day has been associated with variable TH levels [17,18]. For example, a study of 47 patients without previous use of TH admitted with AMI, noted a decrease in total T3 (~19% at 24–26 hours when compared to a sample that was obtained during the first 6 hours) and TSH levels (~51%, when comparing similar timeframes). A more pronounced decrease in T3 levels was associated with higher levels of pro-inflammatory hormones and a worst prognosis [18]. However, data from other small studies have showed inconsistent results in terms of TSH variability, suggesting minor elevation [25,26,27].

A study of 4748 patients with AMI undergoing percutaneous coronary intervention found an increased risk for all cause and cardiac mortality for those found to have elevated TSH levels (including patients with a previous diagnosis of hypothyroidism) when compared to those who were euthyroid [28]. Furthermore, the restoration of euthyroidism in this acute setting has been associated with cardioprotective effects in animal models, including the reduction of the myocardial infarction area, induction of physiological hypertrophy, and positive cardiac remodeling [29,30,31,32]. In fact, TH treatment improves left ventricular function, inhibits the expansion of scar tissue, and might have a direct role in the pathophysiology that is associated with heart failure. Most commonly, in these animal studies, treatment is administered, regardless of thyroid hormone status and early in the evolution of the ischemic injury [29,30,31].

Moreover, the effect and adequate choice of TH replacement is not known. For example, euthyroid sick syndrome is common in patients with AMI and is associated with adverse clinical outcomes [20]. A recent randomized study including 37 patients evaluated the value of low dose T3 treatment for patients with ST elevation MI and low T3 syndrome, started 72 h after hospital admission. No patient in the treatment group developed symptoms of hyperthyroidism or arrhythmias and, in four patients, the T3 dose was decreased during follow up due to elevated T3 levels. Left ventricular function and the extent of MRI cardiac necrosis did not differ between the groups at six months of follow up and a trend towards improved regional contractile dysfunction in those treated was noted [19]. In our study, no patient was started on T3 therapy and T3 values were not commonly measured. It is important to note that our population did not have low T3 syndrome, given the elevated TSH measurements.

Lastly, the modulation of TH has emerged as a potential therapeutic target for patients with AMI in the subacute and chronic setting. Cardio-protection aims to minimize irreversible ischemic damage and it favors functional recovery of the injured myocardium. TH regulates intracellular pro survival pathways, promotes preservation of mitochondrial function, antifibrotic and angiogenic effects, and potentially induces cell regeneration [2,29,30,31]. For example, a prospective study of 102 patients with STEMI and without history of previous LT4 use found that 7% of patients had a TSH value of more than 4 mU/L. Moreover, in this group of mostly euthyroid patients, an adjusted logistic regression analysis showed that lower baseline TSH levels were associated with an increased risk of left ventricular cardiac remodeling, using a baseline TSH value of <1.38 mU/L for comparisons [33]. A randomized study evaluating the effect of treatment of SCH 21 days after AMI is underway and it might provide clinical evidence related to cardio protection from TH treatment for patients with TSH 4–10 mIU/L (measured on day 1 and day 7–10 after the event), in patients without a previous history of LT4 use [34].

Our study is limited by its retrospective design and small sample size. Moreover, only 44% of patients who were admitted with AMI underwent evaluation of their thyroid status, leading to a highly selected population. This provides unstable estimates and decreases our ability to detect differences between groups (and subgroups) and adequately adjust for confounders. However, although animal studies have evaluated the potential risk and benefits of LT4 treatment in the setting of AMI, to our knowledge, no clinical studies in this population have been reported. As such, the comprehensive description presented in this retrospective study provides an initial observation of the clinical features guiding the management of hypothyroidism in the setting of AMI, and the potential safety of treatment with LT4 in the acute setting to restore thyroid function. Moreover, we focused on patients with clinically relevant hypothyroidism, including those with previous history of LT4 use in the setting of AMI, where the impact of LT4 treatment might be more evident when compared to those with milder thyroid dysfunction. Based on our initial observation, the degrees of TSH elevation, type of hypothyroidism, and type of MI are important factors to consider in larger studies addressing the outcomes of MI in the setting of hypothyroidism.

## 5. Conclusions

In patients found to have biochemical evidence of hypothyroidism while being admitted for the management of AMI clinicians are more likely to start/resume treatment with LT4 for those with higher elevation of TSH or a previous history of hypothyroidism. Similarly, IV LT4 seems to be more frequently used in patients with higher TSH levels and those who are more critically ill. Further clinical studies are needed in order to clarify the safety and efficacy of LT4 treatment for hypothyroidism in the setting of AMI.

## Figures and Tables

**Table 1:** Demographics, comorbidities, and risk factors according to thyroid function and treatment status.

		Thyroid Function			Treatment Status		
	All (*n*: 64)	Subclinical Hypothyroidism and High TSH Levels (*n*: 40, 63%)	Overt Hypothyroidism (*n*: 24, 37%)	*p* Value	Yes (*n*: 52, 81%)	No (*n*: 12, 19%)	*p* Value
**Age (Years)**	64 (55–79)	64 (54–80)	64 (56–78)	0.647	63 (55–79)	65 (55–78)	0.836
**Sex (WOMAN)**	61%	65%	54%	0.390	63%	50%	0.514
**Race**	Caucasian 78% African American 17% Hispanic 3% Other 2%	83% 15% 2% 0%	71% 21% 4% 4%	0.353 (Caucasian)	81% 13% 4% 2%	67% 33% 0 0	0.438
**LOS (Days)**	7 (4–14)	7 (4–14)	8 (4–14)	0.840	7 (4–14)	7 (3–14)	0.750
**BMI (kg/m^2^)**	27 (22–32)	28 (23–32)	26 (21–32)	0.363	28 (22–32)	27 (23–31)	0.717
**HTN**	70%	75%	63%	0.289	71%	67%	0.739
**DM**	45%	50%	38%	0.331	48%	33%	0.522
**Smoking (Never)**	22%	25%	17%	0.541	21%	25%	0.715
**Dyslipidemia**	66%	63%	71%	0.497	71%	42%	0.090
**History of CHD**	42%	48%	33%	0.267	48%	17%	0.058
**PVD**	5%	5%	4%	1	6%	0	1
**COPD**	23%	18%	33%	0.226	27%	8%	0.265
**Sleep Apnea**	8%	3%	17%	0.061	10%	0	0.574
**CKD**	28%	35%	17%	0.114	25%	42%	0.293
**History of Stroke**	25%	20%	33%	0.233	25%	25%	1
**Aspirin Use**	36%	33%	42%	0.459	40%	17%	0.185
**History of HF**	31%	35%	25%	0.403	29%	42%	0.493

LOS, length of stay; BMI, body mass index; HTN, hypertension; DM, diabetes mellitus; CHD, coronary heart disease; PVD, peripheral vascular disease; COPD, chronic obstructive pulmonary disease; CKD, chronic kidney disease; HF, heart failure.

**Table 2:** Clinical presentation, laboratory and imaging results according to thyroid function and treatment status.

		Thyroid Function			Treatment Status		
	All (*n*: 64)	Subclinical Hypothyroidism and High TSH Levels (*n*: 40, 63%)	Overt Hypothyroidism (*n*: 24, 37%)	*p* Value	Yes (*n*: 52, 81%)	No (*n*: 12, 19%)	*p* Value
**TSH on Admission** **(mIU/L)**	20 (12–40)	14 (12–22)	42 (28–86)	<0.001	25 (13–43)	12 (11–19)	0.007
**Hemodynamically unstable**	27%	25%	29%	0.715	21%	50%	0.067
**Myxedema Score ≥ 60**	20%	20%	21%	1	17%	33%	0.243
**Pericardial/Pleural Effusion**	34%	35%	33%	0.892	33%	42%	0.737
**Pulmonary Edema**	20%	20%	21%	1	17%	33%	0.243
**Cardiomegaly**	28%	32%	21%	0.315	27%	33%	0.726
**EF ≤ 40%**	41%	43%	38%	0.693	42%	33%	0.747
**STEMI**	16%	15%	17%	1	13%	25%	0.381

TSH, thyroid stimulation hormone; EF, ejection fraction; STEMI, ST segment elevation myocardial infarction. Hemodynamically unstable (based on blood pressure, heart rate and need for pressors) Myxedema score (temperature, central nervous system effects, gastrointestinal findings, precipitating events, cardiovascular dysfunction and metabolic abnormalities). Pericardial/pleural effusion, pulmonary edema, cardiomegaly (based on imaging).

**Table 3:** Clinical variables according to route of initial levothyroxine treatment.

	Route of Levothyroxine Administration		
Clinical Variable	IV (*n*: 8)	PO (*n*: 44)	*p*
**Age (Years)**	65 (53–87)	63 (55–78)	0.639
**Sex (Woman)**	63%	64%	1
**EF < 40%**	63%	39%	0.260
**Myxedema Score > 60**	25%	16%	0.615
**TSH (mIU/L)**	36 (14–87)	23 (13–42)	0.432
**Hemodynamically Unstable**	50%	16%	0.051
**Endocrinology Consulted**	50%	25%	0.207
**STEMI**	0%	16%	0.578

IV, intravenous; po, oral; EF, ejection fraction; TSH, thyroid stimulating hormone, STEMI, ST segment elevation myocardial infarction.

**Table 4:** Concomitant management according to thyroid function and treatment status.

		Thyroid Function			Treatment Status		
	All (*n*: 64)	Subclinical Hypothyroidism or High TSH (*n*: 40, 63%)	Overt Hypothyroidism (*n*: 24, 37%)	*p* Value	Yes (*n*: 52, 81%)	No (*n*: 12, 19%)	*p* Value
**Beta Blocker Use**	81%	80%	83%	1	81%	83%	1
**Antiplatelet**	92%	90%	96%	0.6424	94%	83%	0.2330
**ACEI**	56%	55%	58%	0.7497	63%	25%	0.0233
**ARB**	6%	8%	4%	1	6%	8%	0.5739
**Statin**	83%	85%	79%	0.7336	84%	75%	0.4182
**PCI**	28%	28%	29%	0.8858	29%	25%	1
**CABG**	5%	5%	4%	1	6%	0%	1

Antiplatelet—Aspirin, Clopidogrel, Prasugrel, Ticagrelor, Anticoagulant-Coumadin, Heparin, Low Molecular weight heparin, rivaroxaban, apixaban, dabigatran, TSH, thyroid stimulating hormone; ACEI, angiotensin converting enzyme inhibitor; ARB, angiotensin II receptor blockers; PCI, percutaneous coronary intervention; CABG, coronary artery bypass grafting.

**Table 5:** Clinical outcomes according to treatment status and thyroid function.

Outcomes		Treatment Status			Thyroid Function		
	All	Yes (*n*: 52)	No (*n*: 12)	*p* Value	SCH (*n*: 40)	Overt Hypothyroidism (*n*: 24)	*p* Value
**30 Day Mortality** **(*n*: 64)**	9% (6)	6.0% (3)	25% (3)	0.074	10% (4) (1 treated and 3 not treated)	8% (2) (2 treated)	1
**New Diagnosis or Worsening of Heart Failure** **(*n*: 64)**	28% (18)	25% (13)	42% (5)	0.294	30% (12) (7 treated and 5 not treated)	25% (6) (6 treated)	0.667
		(*n*: 48)	(*n*: 10)		(*n*: 36)	(*n*: 22)	
**30 Day Readmission** **(*n*: 58)**	10% (6)	13% (6)	0%	0.577	11% (4) (4 treated)	9% (2) (2 treated)	1

New diagnosis of heart failure or worsening—evaluated according to changes in ejection fraction or clinical diagnosis.

**Table 6:** Clinical outcomes according to treatment status and timing of levothyroxine treatment.

	Patients with Events	Patient with Events (No LT4 Treatment)	Patients with Events (LT4 Treatment)	*p* Value	Patients with Events (Before LT4)	Patients with Events (After LT4)
**Bleeding**	9% (6/64)	17% (2/12)	8% (4/52)	0.313	4% (2/52)	4% (2/52)
**Stroke**	6% (4/64)	0% (0/12)	8% (4/52)	1	6% (3/52)	2% (1/52)
Arrhythmia *	25% (16/64)	33% (4/12)	23% (12/52)	0.475	15% (8/52)	10% (5/52)

LT4, levothyroxine. * One of the treated patients had arrhythmia before and after levothyroxine treatment. Bleeding-clinical documentation of overt blood loss. Stroke-clinical documentation based on presentation and imaging findings. Arrhythmia-clinical documentation based on presentation and clinical findings.

## Appendix A

### Acute Myocardial Infarction International Classification of Diseases (ICD)

**ICD 9**

410 Acute myocardial infarction

410.0 Acute myocardial infarction of anterolateral wall

410.00 Acute myocardial infarction of anterolateral wall, episode of care unspecified

410.01 Acute myocardial infarction of anterolateral wall, initial episode of care

410.02 Acute myocardial infarction of anterolateral wall, subsequent episode of care

410.1 Acute myocardial infarction of other anterior wall

410.10 Acute myocardial infarction of other anterior wall, episode of care unspecified

410.11 Acute myocardial infarction of other anterior wall, initial episode of care

410.12 Acute myocardial infarction of other anterior wall, subsequent episode of care

410.2 Acute myocardial infarction of inferolateral wall

410.20 Acute myocardial infarction of inferolateral wall, episode of care unspecified

410.21 Acute myocardial infarction of inferolateral wall, initial episode of care

410.22 Acute myocardial infarction of inferolateral wall, subsequent episode of care

410.3 Acute myocardial infarction of inferoposterior wall

410.30 Acute myocardial infarction of inferoposterior wall, episode of care unspecified

410.31 Acute myocardial infarction of inferoposterior wall, initial episode of care

410.31 Acute myocardial infarction of inferoposterior wall, subsequent episode of care

410.4 Acute myocardial infarction of other inferior wall

410.40 Acute myocardial infarction of other inferior wall, episode of care unspecified

410.41 Acute myocardial infarction of other inferior wall, initial episode of care

410.42 Acute myocardial infarction of other inferior wall, subsequent episode of care

410.5 Acute myocardial infarction of other lateral wall

410.50 Acute myocardial infarction of other lateral wall, episode of care unspecified

410.51 Acute myocardial infarction of other lateral wall, initial episode of care

410.52 Acute myocardial infarction of other lateral wall, subsequent episode of care

410.6 True posterior wall infarction

410.60 True posterior wall infarction, episode of care unspecified

410.61 True posterior wall infarction, initial episode of care

410.62 True posterior wall infarction, subsequent episode of care

410.7 Subendocardial infarction

410.70 Subendocardial infarction, episode of care unspecified

410.71 Subendocardial infarction, initial episode of care

410.72 Subendocardial infarction, subsequent episode of care

410.8 Acute myocardial infarction of other specified sites

410.80 Acute myocardial infarction of other specified sites, episode of care unspecified

410.81 Acute myocardial infarction of other specified sites, initial episode of care

410.82 Acute myocardial infarction of other specified sites, subsequent episode of care

410.9 Acute myocardial infarction of unspecified site

410.90 Acute myocardial infarction of unspecified site, episode of care unspecified

410.91 Acute myocardial infarction of unspecified site, initial episode of care

410.92 Acute myocardial infarction of unspecified site, subsequent episode of care

**ICD10**

I21 Acute myocardial infarction

I21.0 Acute transmural myocardial infarction of anterior wall

I21.01 ST elevation (STEMI) myocardial infarction involving left main coronary artery

I21.02 ST elevation (STEMI) myocardial infarction involving left LAD coronary artery

I21.09 ST elevation (STEMI) myocardial infarction involving other coronary artery of anterior wall

I21.1 Acute transmural myocardial infarction of inferior wall

I21.11 ST elevation (STEMI) myocardial infarction involving right coronary artery

I21.19 ST elevation (STEMI) myocardial infarction involving other coronary artery of inferior wall

I21.2 Acute transmural myocardial infarction of other sites

I21.21 ST elevation (STEMI) myocardial infarction involving left circumflex coronary artery

I21.29 ST elevation (STEMI) myocardial infarction involving other sites

I21.3 ST elevation (STEMI) myocardial infarction of unspecified site

I21.4 Non-STelevation (NSTEMI) myocardial infarction

I21.9 Acute myocardial infarction, unspecified

I21.A Other type of myocardial infarction

I21.A1 Myocardial infarction type 2

I21.A9 Other myocardial infarction type

I22 Subsequent myocardial infarction

I22.0 Subsequent myocardial infarction of anterior wall

I22.1 Subsequent myocardial infarction of inferior wall

I22.8 Subsequent myocardial infarction of other sites

I22.9 Subsequent myocardial infarction of unspecified site

### Thyroid Stimulating Hormone (TSH) Current Procedural Terminology (CPT Codes)

80092 Thyroid panel with TSH

84436 Thyroxine, total

84439 Thyroxine Free

84443 Thyroid Stimulating Hormone (TSH)

84480 Total T3

84482 Reverse T3

**Component Test Code**	**Component Chart Name**	**LOINC**
0070225	TSH 3rd Generation	11580-8
